# Surgical Management of Ankle Fracture in Late Pregnancy: A Case Report

**DOI:** 10.7759/cureus.85618

**Published:** 2025-06-09

**Authors:** Taro Kasai, Yumi Ata, Motohiko Hara, Mai Yamoto, Koichi Maruyama, Tetsuro Yasui

**Affiliations:** 1 Department of Orthopaedic Surgery, Faculty of Medicine, University of Tokyo, Tokyo, JPN; 2 Department of Orthopaedic Surgery, Teikyo University Mizonokuchi Hospital, Kawasaki, JPN; 3 Department of Rehabilitation Medicine, Teikyo University Mizonokuchi Hospital, Kawasaki, JPN; 4 Department of Anesthesiology, Teikyo University Mizonokuchi Hospital, Kawasaki, JPN

**Keywords:** ankle fracture, late pregnancy, multidisciplinary team approach, obstetrics, surgery

## Abstract

Surgical management of ankle fractures during late pregnancy is rare and presents a unique challenge. We report the case of a 40-year-old woman at 30 weeks of gestation who sustained an unstable trimalleolar ankle fracture and was successfully treated with surgery without the need for immediate cesarean section. The fracture surgery was performed under combined spinal and epidural anesthesia with continuous fetal heart monitoring. The patient was placed in the left lateral decubitus position to avoid inferior vena cava compression. Low-dose fluoroscopy with abdominal shielding minimized fetal radiation exposure. To prevent deep vein thrombosis, a tourniquet was not used, preoperative venous ultrasonography was conducted, and postoperative anticoagulation was administered. Acetaminophen was used for pain control to avoid the risk of fetal ductal constriction associated with nonsteroidal anti-inflammatory drugs. Postoperative rehabilitation focused on safe mobilization while minimizing intra-abdominal pressure. This case highlights the importance of a multidisciplinary team approach in managing orthopedic surgeries during late pregnancy to achieve both maternal fracture healing and full-term vaginal delivery of the fetus.

## Introduction

Trauma during pregnancy is reported to occur in approximately 8% of all pregnancies and remains the leading cause of non-obstetric maternal death in the United States [1]. Ankle fractures are common orthopedic injuries that affect individuals across a wide range of ages and backgrounds [2]. During pregnancy, ankle fractures account for approximately 39% of fractures resulting from low-energy trauma [3]. However, surgical management of ankle fractures in pregnant patients, particularly in late pregnancy, has rarely been documented [4-6]. The treatment of orthopedic trauma during late pregnancy requires a careful balance between ensuring maternal fracture healing and fetal well-being [7].

In this report, we present the case of a patient in late pregnancy who sustained an ankle fracture that was successfully treated with surgical intervention without the need for cesarean section. To date, only a few reports have described the surgical management of ankle fractures during late pregnancy [4-6]. Of these, one case involved fracture surgery performed alone [5]. In another case, a planned cesarean section was performed concurrently with the fracture surgery [6], while in the other, an emergent cesarean section was necessitated due to fetal heart rate abnormalities observed during the fracture surgery [4]. Therefore, individualized treatment strategies tailored to the patient's specific condition are crucial in managing ankle fractures during late pregnancy. This case report provides a detailed description of the perioperative management and multidisciplinary collaboration among healthcare professionals.

This article was previously presented as a meeting abstract at the 48th Annual Meeting of the Japanese Society for Surgery of the Foot on October 26, 2023.

## Case presentation

**Figure 1:**
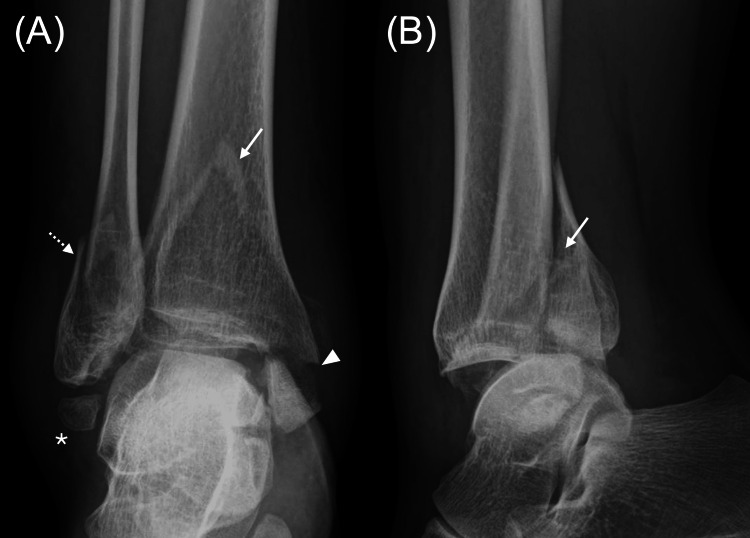
Plain radiographs of the right ankle in (A) anteroposterior and (B) lateral views before fracture surgery A displaced posterior malleolar fracture involving approximately 40% of the articular surface (solid arrows) is seen, along with displaced fractures of the lateral (dashed arrow) and medial malleoli (arrowhead), and a naturally occurring os subfibulare located inferior to the lateral malleolus (asterisk).

A 40-year-old pregnant woman at 30 weeks of gestation was referred to our institution with severe right ankle pain following a fall. The right ankle showed marked swelling. There were no signs of circulatory impairment or motor-sensory deficits distal to the ankle. Radiographic evaluation revealed a displaced posterior malleolar fracture involving approximately 40% of the articular surface along with displaced fractures of the lateral and medial malleoli (Figure 1). In addition, the os subfibulare was found to be inferior to the lateral malleolus. Computed tomography was not performed to avoid fetal radiation exposure. Magnetic resonance imaging was not performed due to the risk of inferior vena cava syndrome, which can occur in pregnant women during prolonged supine positioning in late pregnancy. The patient was diagnosed with a trimalleolar ankle fracture requiring surgery.

The obstetrician confirmed that the maternal fall trauma had no adverse effects on the fetus. Considering the gestational age, cesarean delivery was deemed too early, and surgical management of the fracture was prioritized.

**Figure 2:**
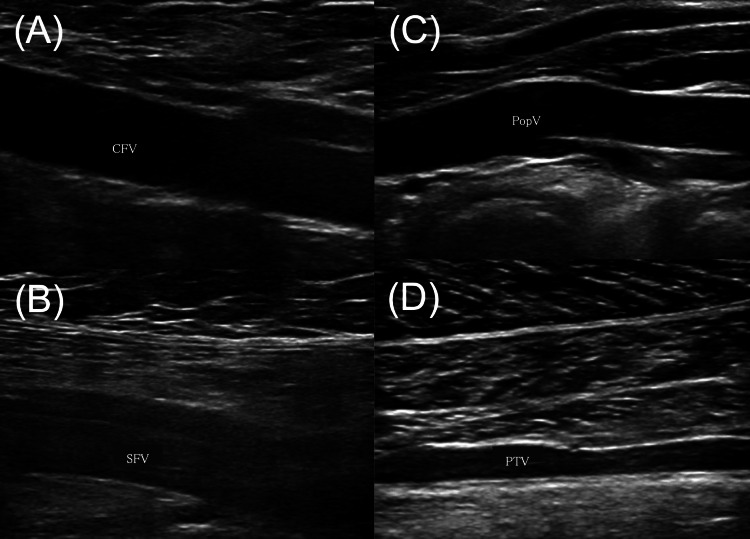
Lower-extremity venous ultrasound images before fracture surgery, demonstrating no deep vein thrombosis (A) Common femoral vein; (B) Superficial femoral vein; (C) Popliteal vein; (D) Posterior tibial vein

Preoperative discussions with anesthesiologists were conducted to determine the optimal anesthesia strategy, intraoperative positioning, and contingency planning for emergency cesarean section, if necessary. Given the increased risk of deep vein thrombosis (DVT) during pregnancy, a preoperative lower-extremity venous ultrasound was performed, confirming the absence of DVT (Figure 2). Acetaminophen was used for pain management to avoid the risk of fetal ductal constriction associated with nonsteroidal anti-inflammatory drugs (NSAIDs). After meticulous perioperative planning with the multidisciplinary team, fracture surgery was performed two days after the injury.

In the operating room, fetal heart rate monitoring was used for continuous fetal assessment. To minimize the effects of anesthetic agents on fetal heart rate variability, a combination of spinal and epidural anesthesia was administered. Additionally, to ensure adequate fetal perfusion and prevent inferior vena cava compression, the patient was placed in the left lateral decubitus position. The maternal abdomen was fully shielded with a radiation-protective barrier, and low-dose fluoroscopy was used to minimize radiation exposure.

**Figure 3:**
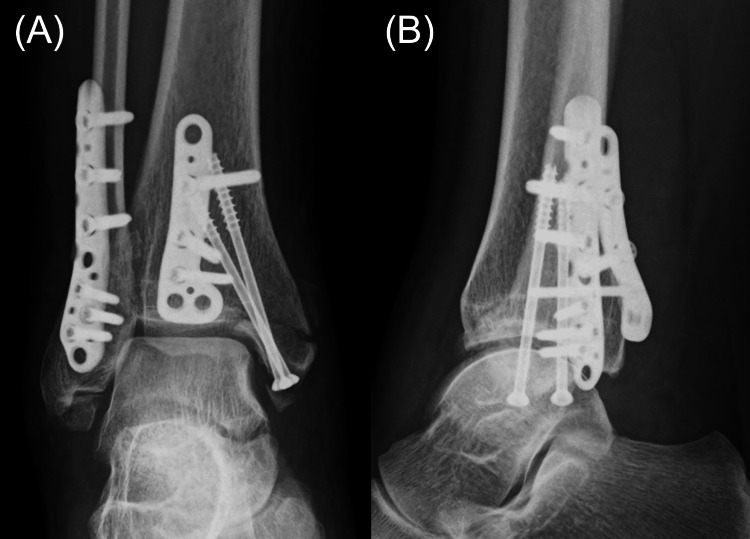
Plain radiographs of the right ankle in (A) anteroposterior and (B) lateral views immediately after fracture surgery The posterior and lateral malleolar fragments can be seen fixed with a plate and the medial malleolar fragment is seen fixed with screws.

To minimize the risk of DVT, a tourniquet was not used. A posterolateral approach was used to fix the large posterior malleolar Volkmann fragment and the lateral malleolar fragment with plates. A medial approach was used to secure the medial malleolar fragment with two screws (Figure 3). Careful dissection was performed to prevent injury to the critical anatomical structures, including the sural and peroneal tendons. Throughout the procedure, the fetal heart rate remained stable, and the surgery was completed without complications. The operative time was 120 minutes, and blood loss was minimal.

Postoperatively, the ankle was immobilized with a splint for one week, followed by restricted weight-bearing for four weeks. Ankle range-of-motion exercises were initiated one week after surgery, and full weight-bearing was permitted at four weeks postoperatively. Considering the high risk of DVT during pregnancy, thromboprophylaxis with subcutaneous heparin calcium was administered along with early mobilization. NSAIDs continued to be avoided to prevent fetal ductal constriction. Rehabilitation focused on cautious gait training while minimizing intra-abdominal pressure. By postoperative week 6, the patient achieved independent ambulation.

**Figure 4:**
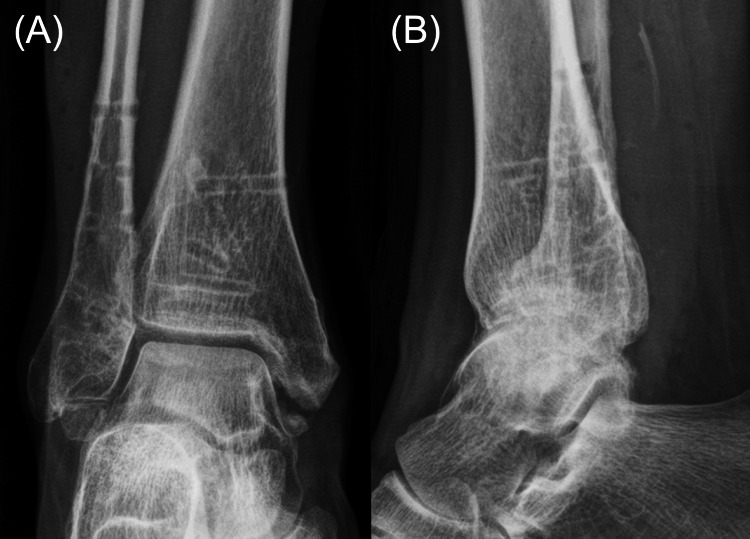
Plain radiographs of the right ankle in (A) anteroposterior and (A) lateral views at the 20-month final follow-up after fracture surgery demonstrating bone union without post-traumatic osteoarthritis

The postoperative course was uneventful with no adverse effects on the mother or fetus. At 40 weeks of gestation, the patient vaginally delivered a healthy infant. Eighteen months after fracture surgery, implant removal surgery was performed based on the patient's preference, although no implant-related adverse events were noted. At the 20-month final follow-up after fracture surgery, radiographic examination confirmed bone union without post-traumatic osteoarthritis (Figure 4). The patient exhibited no pain or restrictions in ankle range of motion, and no postpartum complications related to the fracture were observed. The patient’s child achieved normal developmental milestones.

## Discussion

Surgical management of traumatic injuries during late pregnancy requires a careful balance between maternal care and fetal well-being. This case demonstrates how coordinated multidisciplinary planning and individualized perioperative management can result in favorable outcomes.

One of the primary challenges is determining whether fracture surgery should proceed independently or be combined with delivery. In one reported case, the fracture surgery and planned cesarean section were performed together at 38 weeks of gestation, which is classified as full term, and no postoperative complications occurred [6]. In the other reported case, the fracture surgery was initially performed independently at 36 weeks of gestation, which is considered late preterm, but fetal heart rate decelerations occurred during the procedure and an emergency cesarean section was required [4]. Although our patient was in a later stage of pregnancy, she was only at 30 weeks of gestation, which is considered too early for a cesarean section. Therefore, we decided to perform fracture surgery independently, aiming for full-term birth.

The selection of the appropriate surgical position is another significant challenge. Supine positioning during late pregnancy poses the risk of inferior vena cava syndrome due to compression of the inferior vena cava by the enlarged uterus, leading to reduced fetal blood flow [8]. The left lateral decubitus position is recommended, as it alleviates the compression of the inferior vena cava by the gravid uterus and minimizes the reduction in venous return to the right atrium. Our patient had a right ankle fracture, and positioning the patient in the left lateral decubitus position to prevent inferior vena cava syndrome did not interfere with the surgery. However, in cases of left ankle fractures, the left lateral decubitus position may impede surgical maneuvers, necessitating modifications to surgical positioning. A previously reported case of emergency cesarean section during ankle fracture surgery involved a left ankle fracture [4], underscoring the importance of careful consideration of surgical positioning during late pregnancy.

Anesthesia selection requires careful balancing of maternal comfort and fetal safety. Spinal and epidural anesthesia for lower abdominal and lower extremity surgery in pregnant patients has been reported to be associated with relatively low drug exposure, minimal impact on fetal heart rate variability, enhanced postoperative analgesia, and early mobilization [9]. The effectiveness of popliteal sciatic nerve block for foot and ankle surgery during late pregnancy has also been reported [10]. Considering the potential for technical failure of the sciatic nerve block, we selected combined spinal and epidural anesthesia.

Another critical concern was intraoperative fetal monitoring. Although there is no established consensus regarding the implementation of continuous intraoperative fetal heart monitoring, a case report documented the detection of fetal distress during ankle fracture surgery via intraoperative monitoring, enabling emergency cesarean section to prevent adverse outcomes [4]. This case suggests that intraoperative fetal heart monitoring may be beneficial in preventing complications in patients undergoing ankle fracture surgery during late pregnancy. Therefore, we performed the surgery with concurrent fetal heart rate monitoring.

Radiation exposure during orthopedic surgery is particularly concerning in pregnant patients. The National Council on Radiation Protection and Measurements recommends that the cumulative fetal radiation dose during pregnancy should not exceed 50 mGy [11,12]. Whenever possible, abdominal and pelvic shielding should be employed to minimize fetal radiation exposure [13]. The primary radiation source of the C-arm fluoroscopy unit is located below the surgical table, and appropriate shielding in this area effectively reduces radiation exposure. Low-dose-mode fluoroscopy can be utilized effectively in cases of ankle fractures in which the soft tissue is relatively thin. In the present case, in collaboration with radiologic technologists, low-dose fluoroscopy and minimization of the number of exposures successfully reduced the total radiation dose to 0.2 mGy.

The prevention of DVT is particularly important because of pregnancy-related physiological changes, including increased blood coagulation and elevated venous pressure [14]. This risk is further exacerbated in cases requiring postoperative immobilization, where the odds ratio for DVT is reported to be 7.7 [15]. Although the incidence of DVT after ankle fracture is generally low, pregnancy-associated risks necessitate proactive preventive strategies, particularly in cases involving ankle fracture surgery during late pregnancy. We performed preoperative venous ultrasonography to confirm the absence of a preexisting thrombosis. Additionally, postoperative anticoagulation therapy was performed with subcutaneous heparin calcium, an unfractionated heparin with a large molecular structure that prevents placental transfer that is considered a safe anticoagulant option during pregnancy [16]. Early ambulation and range-of-motion exercises were also performed postoperatively to reduce the risk of DVT.

Postoperatively, pain management and rehabilitation were carefully tailored to minimize the fetal risk. NSAIDs were avoided because of their potential to induce fetal ductal constriction [17], and we used acetaminophen. Physiotherapy was designed to facilitate the gradual progression of weight-bearing activities and range-of-motion exercises while avoiding excessive intra-abdominal pressure. This individualized rehabilitation program ensured functional recovery for the mother as well as fetal safety.

The specific challenges of orthopedic surgery during pregnancy, as detailed in this case report, need to be carefully evaluated on a case-by-case basis. For each identified challenge, appropriate strategies should be planned before surgery.

## Conclusions

Isolated ankle fracture surgery was performed at 30 weeks of gestation without the need for cesarean section, resulting in both successful full-term vaginal delivery and maternal fracture healing. In managing fracture surgery during late pregnancy, careful consideration should be given to intraoperative positioning, anesthesia selection, minimization of radiation exposure, perioperative DVT prophylaxis, and pain control. Multidisciplinary collaboration among healthcare professionals is essential to achieving favorable outcomes.
